# Community NGO intervention for Alzheimer's patients rehabilitation

**DOI:** 10.1002/alz70860_096311

**Published:** 2025-12-23

**Authors:** S Pal

**Affiliations:** ^1^ SFCCP, meerut, India

## Abstract

**Background:**

Alzheimer's patients Rehabilitation services aren't well‐developed concept in resource‐Poor nations due to poor economics & lack of expertise. With limited resources we wanted to explore these rehabilitation services in rural villages.

**Aims:**

Rural/tribal areas of India lung Alzheimer's patients [Ca] patients lack rehabilitation support available only in city hospitals. Our 12‐year CBO is training youth volunteers & traditional faith healers in marginalized communities to provide rehabilitation.

**Methods:**

Around 1340 cases of Alzheimer's will be in rural/tribal of India by 2025, but hospitals lack dedicated Rehabilitation program. patients returning to villages after onco‐therapy in cities urgently need Rehabilitation support to suit their economic, social, cultural background. Since August 2018 Our NGO clinic started free advise on Rehabilitation. [n = 91]. With 6 trained nurses, two physicians, we screened patients. Average age 62 years, M:F ratio 75:25. Our support services devised as‐per need of Patients in consultation with family & stretched over 21 months included conselling, physiotherapy, occupational therapy, rehabilitation. Responses assessed with Quality of life scale by Flanagan Quality of Life Scale (QOLS) & psychological evaluation by Likert psychometric scale. This presentation demonstrates project in developmental stages & basic operational facts. Initially started as practically oriented guidance center & later developed in well‐organized set‐up offering free rehabilitation services in villages. It comprises NGO staff and traditional faith healers.

**Results:**

Our program highlights variability of psychological & Socio‐medical aspects in rehabilitation. Patents needed medical interventions in 18%, psychological approaches in 84% while remaining 16% needed combination of both.

**Conclusion:**

This is pilot project running on community donation. We plan to document, evaluate this in large sample size, but resource restriction didn't permit. To overcome this, we plan networking of European NGO's, advocacy experts at AAIC‐2023 Venue for better study design, technical support & collaborative efforts. I do not claim “I transformed whole community of patients but we had certainly taken initiative by this cost‐effective approach”.

